# Supplementary material for: Evaluation of therapeutic PD-1 antibodies by an advanced single-molecule imaging system detecting human PD-1 microclusters
Source: Nat Commun. 2023 Jun 6;14:3157. doi: 10.1038/s41467-023-38512-7 (PMC10244369; doi:10.1038/s41467-023-38512-7)
Supplement: Supplementary file 1 — Supplementary Information [file 41467_2023_38512_MOESM1_ESM.pdf]

Supplementary Information

Evaluation of therapeutic PD-1 antibodies by an advanced single-molecule imaging system detecting human PD-1 microclusters

Author list

Wataru Nishi[1, 2], Ei Wakamatsu[2], Hiroaki Machiyama[2], Ryohei Matsushima[1, 2], Kensho Saito[2, 3], Yosuke Yoshida[2, 4], Tetsushi Nishikawa[2, 5], Tomohiro Takehara[6], Hiroko Toyota[2], Masae Furuhata[2], Hitoshi Nishijima[2], Arata Takeuchi[2], Miyuki Azuma[7], Makoto Suzuki[1], and Tadashi Yokosuka[2*]

Affiliations

[1]Department of Thoracic Surgery, Graduate School of Medical Sciences, Kumamoto University, Kumamoto 860-8556, Japan

[2]Department of Immunology, Tokyo Medical University, Tokyo 160-8402, Japan

[3]School of Life Sciences, Tokyo University of Pharmacy and Life Sciences, Tokyo 192-0392, Japan

[4]Department of Nephrology, Tokyo Medical University, Tokyo 160-8402, Japan

[5]Department of Dermatology, Tokyo Medical University, Tokyo 160-0023, Japan

[6]Division of Pulmonary Medicine, Department of Medicine, Keio University School of Medicine, Tokyo 160-8582, Japan

[7]Department of Molecular Immunology, Graduate School of Medical and Dental Sciences, Tokyo Medical and Dental University, Tokyo 113-8549, Japan

Corresponding Author: [*]Tadashi Yokosuka

E-mail: yokosuka@tokyo-med.ac.jp

Supplementary Figure

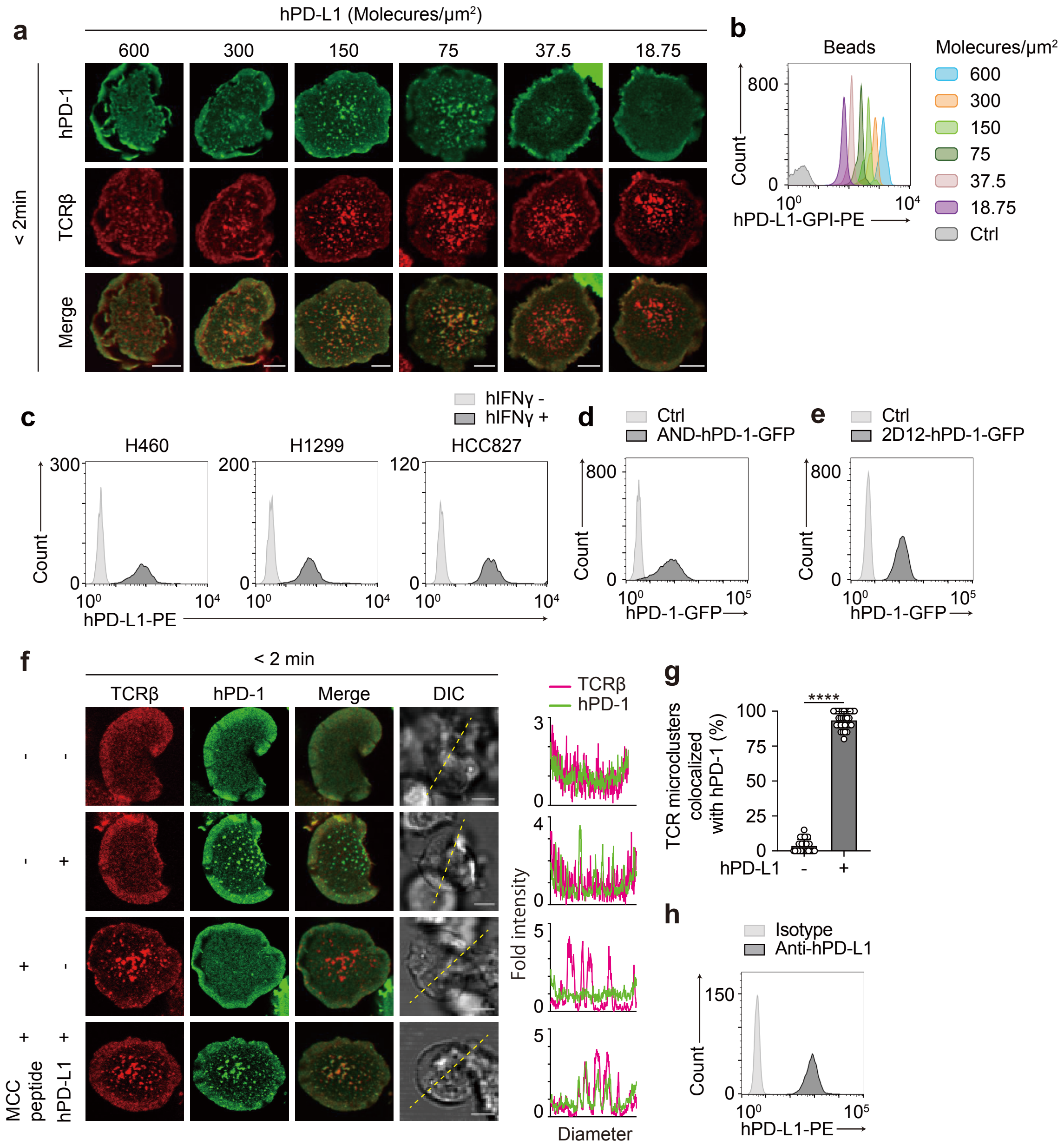

Supplementary Fig. 1

a, 2D12 cells expressing hPD-1-EGFP (green) were prestained with DyLight 650–labeled H57 Fab (red), plated on an SLB containing I-E^{k}–, mICAM-1–, and hPD-L1–GPI (bottom) at the indicated densities as in **Fig. 1a**, and real-time imaged by confocal microscopy 2 min after contact. **b,** Silica beads were coated by lipid-bilayers containing hPD-L1-GPI at the indicated densities. The expression of hPD-L1 on the beads was analyzed by FACS with PE-labeled anti-hPD-L1. **c,** Human lung cancer cell lines, H460,

H1299, and HCC827, were stimulated with hIFNγ at a concentration of 10 ng/mL for 48 hours. The cell surface expression of hPD-L1 was analyzed by FACS with PE-labeled anti-hPD-L1. **d,** AND TCR-Tg $CD4^+$ T cells were transduced with hPD-1-EGFP. The expression of hPD-1-EGFP was analyzed by FACS. **e,** The cells in **a** were analyzed the expression of hPD-1-GFP by FACS. **f,** As in **a**, the cells were imaged on an SLB not prepulsed (top two rows) or prepulsed by $MCC_{88\text{-}103}$ (bottom two rows) without (row 1 and 3) or with hPD-L1–GPI (row 2, 4). TCRβ, red; hPD-1, green. Histograms show fold fluorescent intensities of TCRβ (magenta) and hPD-1 (green) on the diagonal yellow lines in the DIC images. **g,** The graph shows the percentage of TCR microclusters colocalized with hPD-1 2 min after contact in T cells in (**f**, bottom 2 rows) (n = 30). **h,** DC-1 cells were transduced with hPD-L1-HaloTag. The expression of hPD-L1 on DC-1 cells was analyzed by FACS with PE-labeled anti-hPD-L1. All data are representative from two independent experiments. Bars, 5 μm. Data are presented as mean values ± SD. Statistical analysis was performed by an unpaired two-sided *t*-test. ****$p < 0.0001$. Source data for **b-h** are provided as a Source Data file.

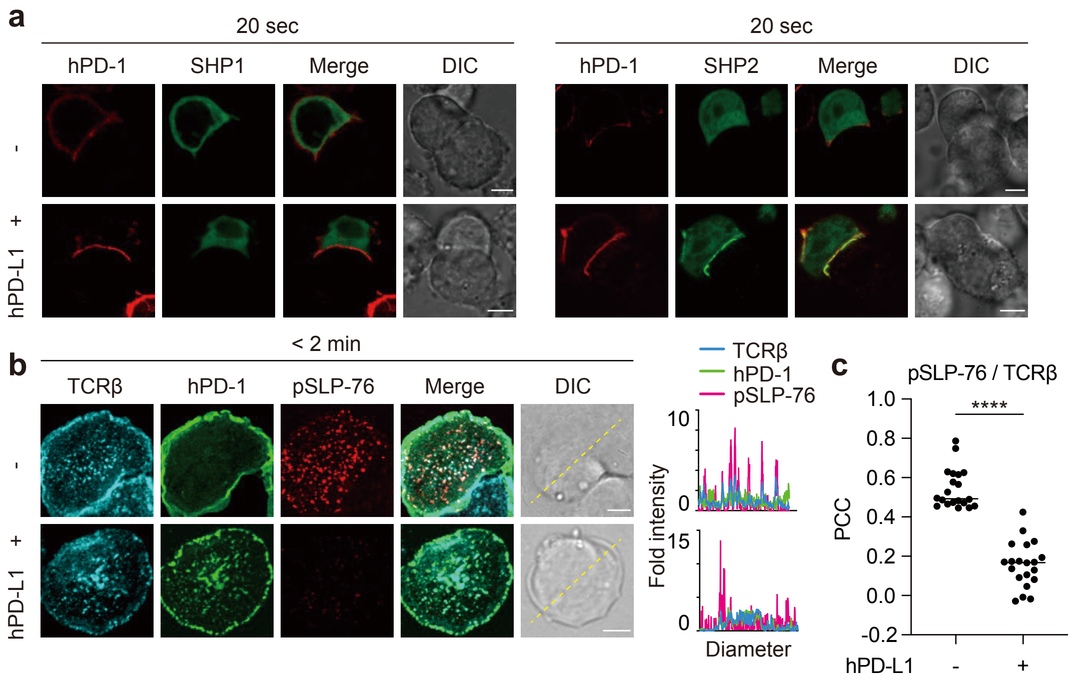

Supplementary Fig. 2

a, As in **Fig. 2a**, 2D12 cells expressing both hPD-1–HaloTag (red) and EGFP–SHP1 (left, green) or –SHP2 (right, green) were conjugated with $MCC_{88\text{-}103}$ prepulsed (1 μM) DC-1 cells not expressing (top) or expressing (bottom) hPD-L1-HaloTag and imaged by confocal microscopy 2 min after T cell-APC contact. **b,** 2D12 cells expressing hPD-1–EGFP (green) were prestained with DyLight 549-labeled H57 Fab (cyan), plated onto an SLB as in Figure 1a without (top) or with hPD-L1–GPI (bottom), fixed 2 min after contact, stained with Alexa Fluor 647–labeled anti-pSLP-76 (red), and imaged by confocal microscopy. Histograms show fold fluorescent intensities of TCRβ (cyan), hPD-1 (green), and p SLP-76 (magenta) on the diagonal yellow lines in the DIC images. **c,** A graph shows the scatter plot summarizing the PCC values in **b**. PCC was calculated between pSLP-76/TCRβ in the absence (left, 0.5430 ± 0.05, mean ± standard deviation [SD]) or presence of hPD-L1–GPI (right, 0.1567 ± 0.05, mean ± SD) by 20 randomly plotted profiles on 20 cells. All data are representative from two independent experiments. Bars, 5 μm. Error bars, SD. Statistical analysis was performed by an unpaired two-sided *t*-test. ****$p < 0.0001$. Source data for **b** and **c** are provided as a Source Data file.

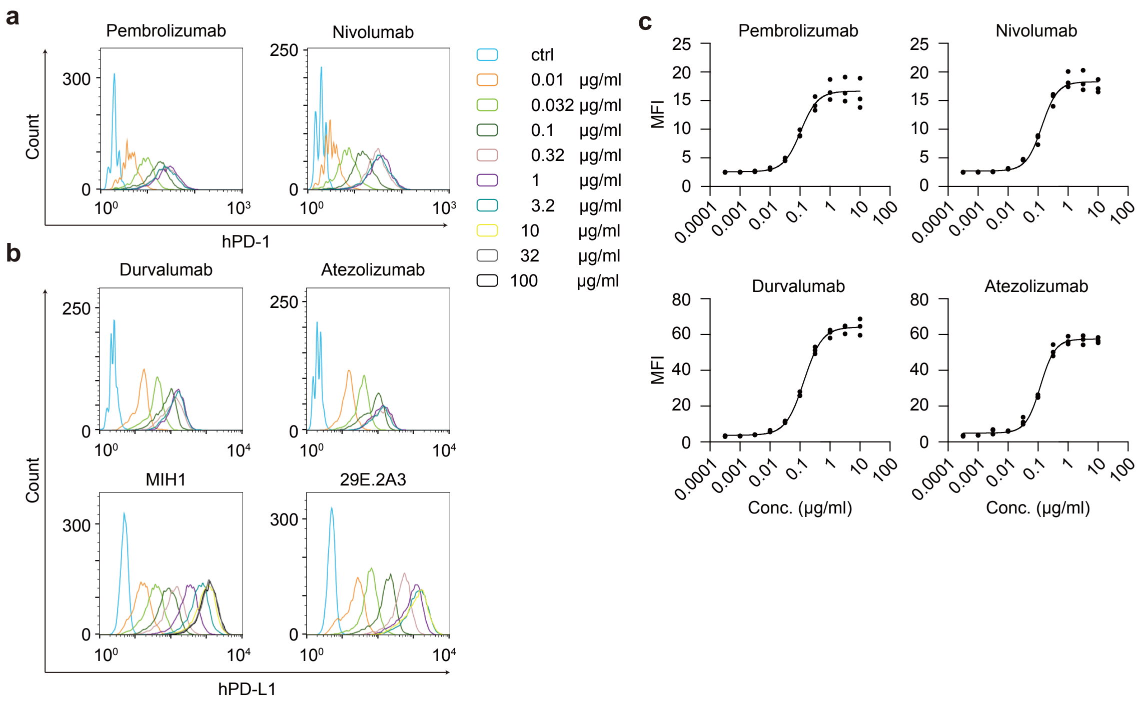

Supplementary Fig. 3

a, 2D12 cells expressing hPD-1 were incubated on ice for 40 mins with human anti-hPD-1, pembrolizumab or nivolumab, at the indicated concentrations, before adding the secondary antibodies, APC-labelled anti-human IgG, and analyzing by FACS to determine the binding capacities of each anti-hPD-1 against 2D12 cells expressing hPD-1. **b,** Similar experiments were performed with DC-1 cells expressing hPD-L1 to examine the avidity of each anti-hPD-L1, durvalumab, atezolizumab, MIH1 or 29E.2A3. The data are representative of three independent experiments performed in triplicate. **c,** The MFI values from FACS data in **a** and **b** are normalized among four anti-hPD-1 and anti-hPD-L1. The binding curve in the presence of each antibody was depicted by a 4-parameter logistic function. Each plot is the data of individual experiments. Source data are provided as a Source Data file.

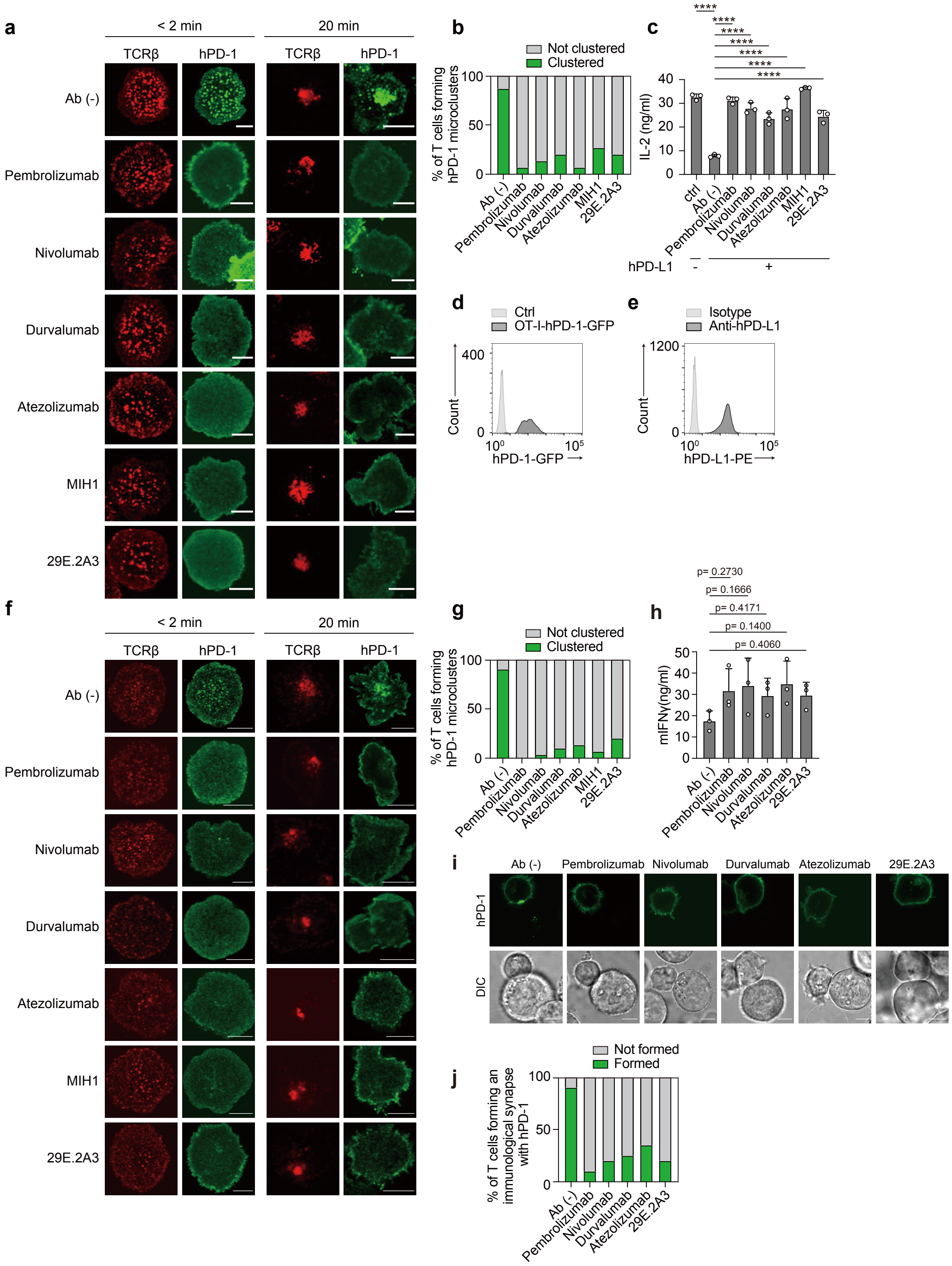

Supplementary Fig. 4

a, AND TCR-Tg CD4$^+$ T cells expressing hPD-1–EGFP (green) were prestained with anti-TCRβ (H57) Fab (red), plated onto an SLB as in **Fig. 1a**, and imaged 2 (left) or 20

min after contact (right) in the absence or presence of the indicated antibodies (10 μg/mL). **b,** The percentage of T cells forming hPD-1 microclusters in **a** (n = 15). **c,** The cells in **a** were cocultured with DC-1 cells not expressing or expressing hPD-L1 in the absence or presence of the indicated antibodies and the concentration of IL-2 was measured by enzyme-linked immunosorbent assay (ELISA) in triplicate. **d,** OT-I TCR-Tg $CD8^+$ T cells were transduced with hPD-1-EGFP. The expression of hPD-1-EGFP was analyzed by FACS. **e,** EL-4 cells were transduced with hPD-L1-HaloTag. The expression of hPD-L1 on EL-4 cells was analyzed by FACS with PE-labeled anti-hPD-L1. **f,** OT-I TCR-Tg $CD8^+$ T cells expressing hPD-1–EGFP (green) were prestained with anti-TCRβ (H57) Fab (red), plated onto an SLB and imaged in the absence or presence of the indicated antibodies (10 μg/mL) as in **a**. **g,** The the percentage of T cells forming hPD-1 microclusters in **f** (n = 30). **h,** OT-I TCR-Tg $CD8^+$ T cells expressing hPD-1–EGFP were cocultured with HCC827 cells expressing $H\text{-}2K^b$ and stimulated with hIFNγ in the absence or presence of each antibody (10 μg/mL). Concentration of mIFNγ was measured by ELISA in triplicate. **i,** OT-I TCR-Tg $CD8^+$ T cells expressing hPD-1–EGFP (green) were conjugated with $OVA_{257\text{-}264}$-prepulsed HCC827 cells, expressing $H\text{-}2K^b$ and stimulated with hIFNγ, in the absence or presence of the indicated antibodies (10 μg/mL) and imaged 2 min after contacts. **j,** The percentages of the cells accompanied by the accumulation of hPD-1 at a T cell-HCC827 cell interface (n = 20). All data are representative of two independent experiments. Bars, 5 μm. Data are presented as mean values ± SD. Statistical analysis was performed by one-way ANOVA. ****$p < 0.0001$. Source data for **b-e**, **g**, **h** and **j** are provided as a Source Data file.

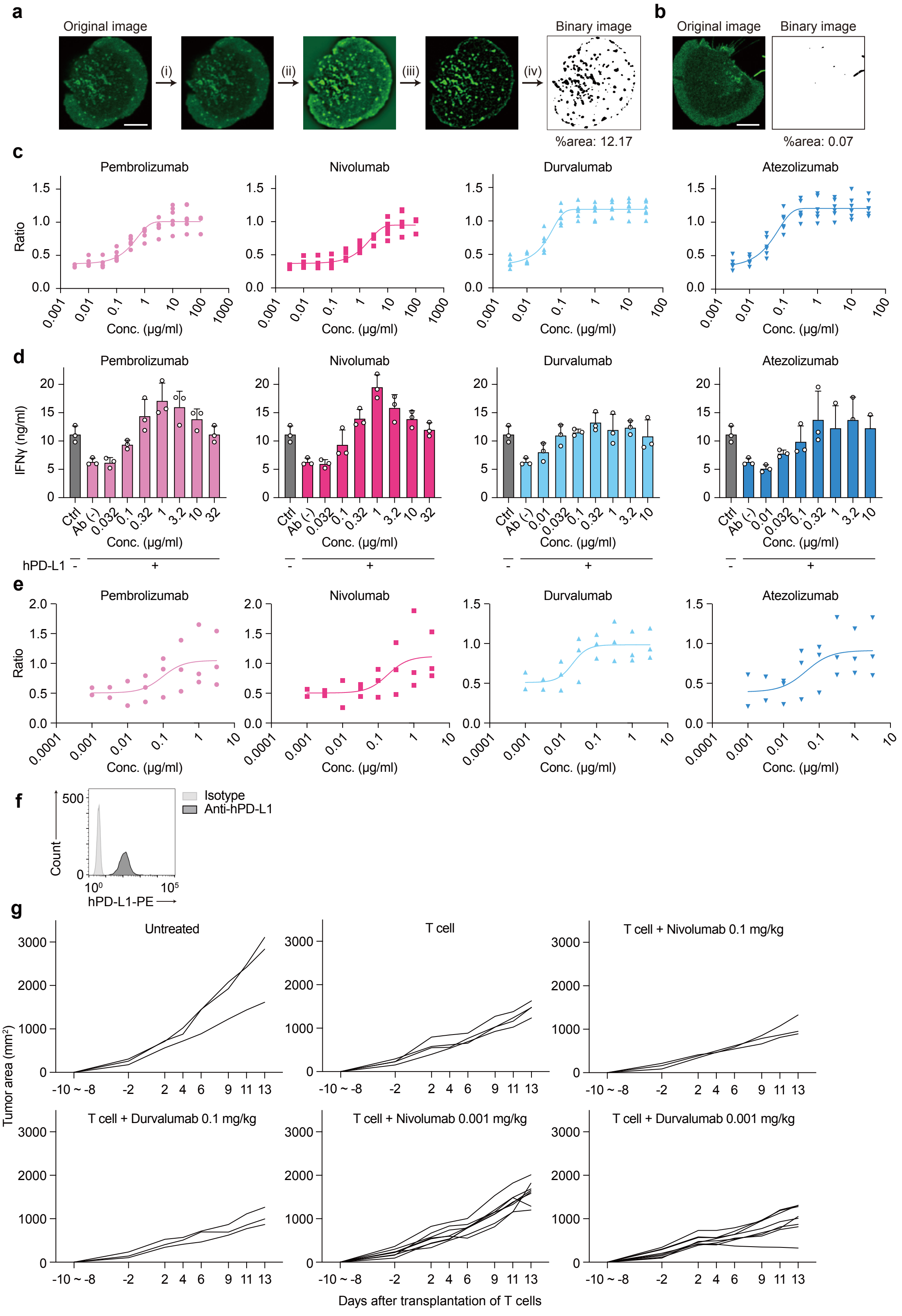

Supplementary Fig. 5

a, Acquired images were applied with 2 times smoothing filter (i), the fast Fourier transform bandpass filter (ii) and the background subtraction using the rolling ball algorithm (iii), and then converted to the binary images with watershed segmentation (iv). The ratio of the total area of microclusters inside T cells (%area) was calculated by dividing the summation of the extracted dark areas in the binary image by the total cell area. **b,** An example image of T cells not forming microcluster is shown, corresponding low value of %area compare to the image in **a**. **c,** The data for IL-2 concentrations of the supernatant in **Fig. 4c** were normalized among four anti-hPD-1 and anti-hPD-L1. The T cell response curve in the presence of each antibody was depicted by a 4-parameter logistic function. Each plot is the data of individual experiments. **d,** The concentration of IFNγ in each supernatant in **Fig. 4g** was measured by ELISA in triplicate. **e,** The data for IFNγ concentrations in **d** were normalized among four antibodies, anti-hPD-1 or anti-hPD-L1. The T cell response curve in the presence of each antibody was depicted by a 4-parameter logistic function. Each plot is the data of individual experiments. **f,** E.G7 cells were transduced with hPD-L1-HaloTag. The expression of hPD-L1 on E.G7 cells was analyzed by FACS with PE-labeled anti-hPD-L1. **g,** Tumor growth curves of the individual mice in **Fig. 4i**. All data are representative from two independent experiments. Bars, 5 μm. Data are presented as mean values ± SD. Source data for **c-g** are provided as a Source Data file.

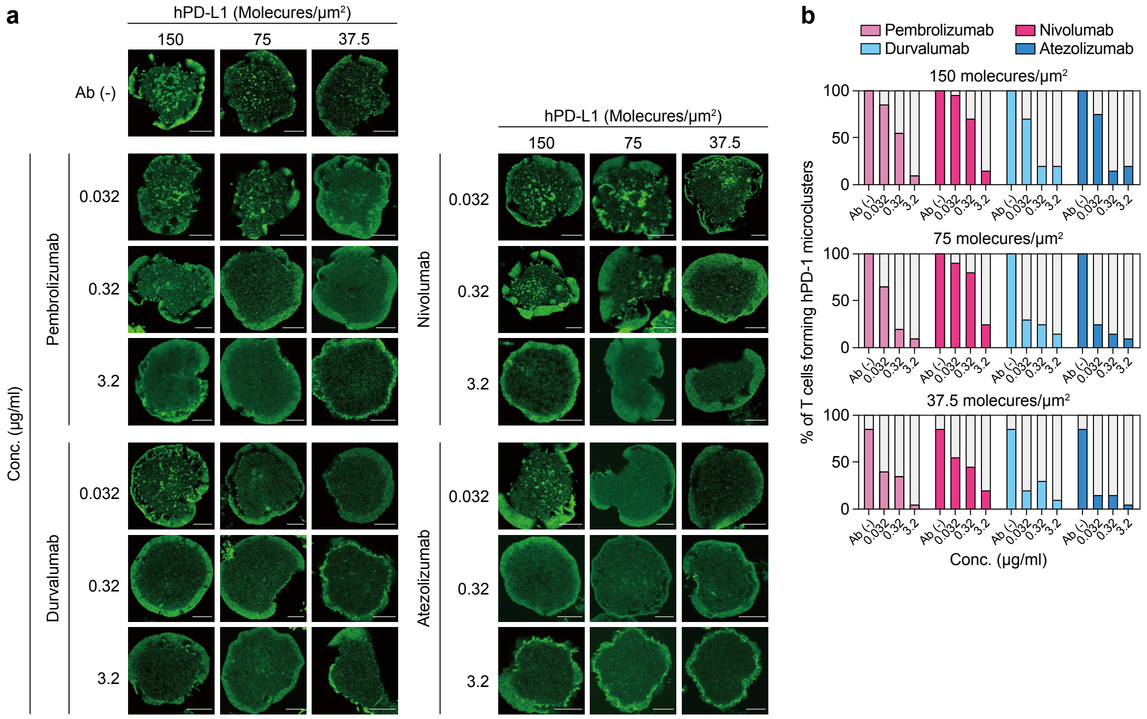

Supplementary Fig. 6

a, 2D12 cells expressing hPD-1-EGFP (green) were plated on an SLB with hPD-L1–GPI at the indicated densities as in **Fig. 1a**. The cells were imaged by confocal microscopy 2 min after contact with each antibody at the indicated concentrations. **b,** The graph shows the percentages of T cells forming hPD-1 microclusters in **a** (n = 20). Bars, 5 μm. All data are representative from two independent experiments. Source data for **b** are provided as a Source Data file.

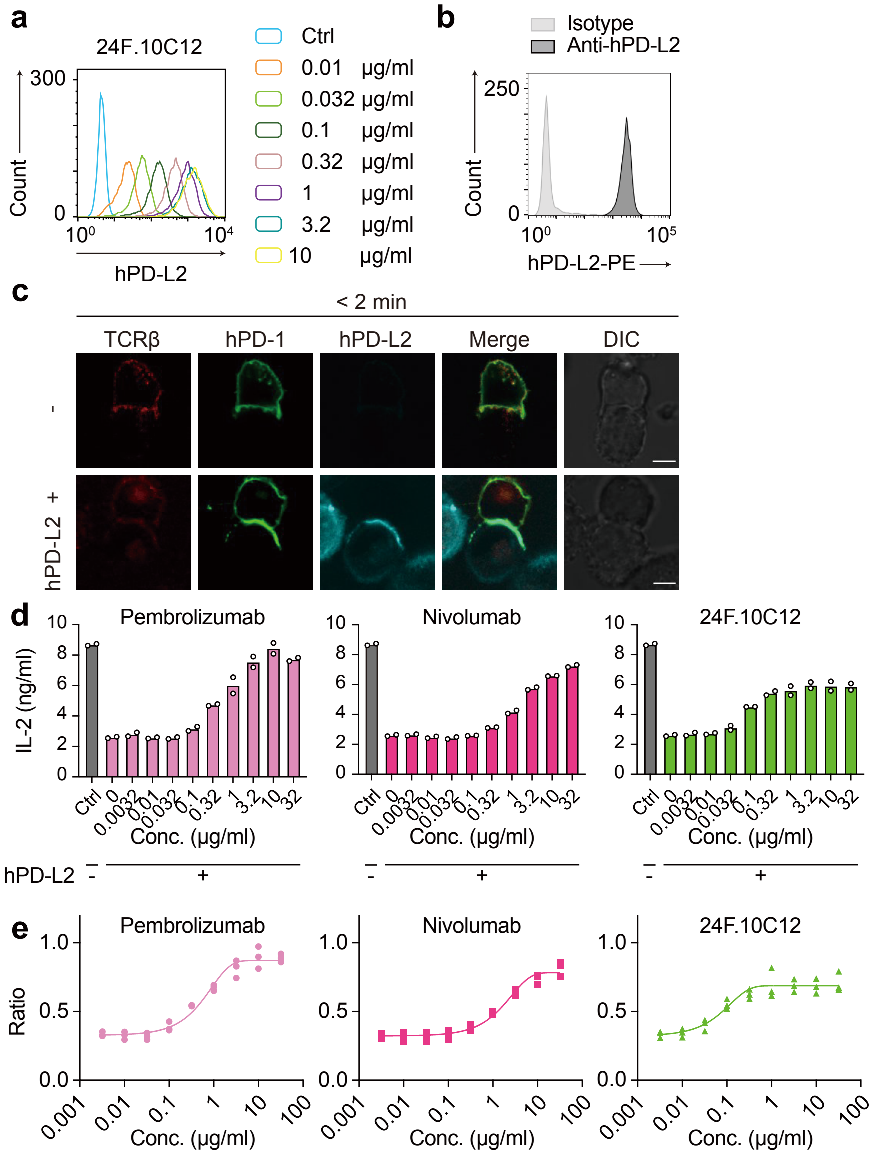

Supplementary Fig. 7

A, DC-1 cells expressing hPD-L2 were incubated on ice for 40 min with anti-hPD-L2, 24F.10C12, at the indicated concentrations, stained by secondary antibodies, APC-labelled anti-human IgG, and analyzed by FACS to determine the binding capacities of 24F.10C12 against DC-1 cells expressing hPD-L2. **B,** The cells in **a** were analyzed for the expression of hPD-L2 by FACS with PE-labeled anti-hPD-L2. **C,** 2D12 cells expressing hPD-1–EGFP (green) were prestained with DyLight 650–labeled H57 Fab (red), conjugated with an $MCC_{88\text{-}103}$ prepulsed (1 µM) DC-1 cells not expressing (top) or expressing (bottom) hPD-L2-HaloTag (cyan) and imaged by confocal microscopy 2 min after T cell-APC contact. **D,** The cells in **c** were cocultured for 16 h with 1 µM

MCC_{88-103} and DC-1 cells not expressing or expressing hPD-L2 in the absence or presence of each antibody at the indicated concentrations and the concentration of IL-2 in each supernatant was measured by ELISA in duplicate. **e,** The data for IL-2 concentrations in **d** were normalized among three antibodies, anti-hPD-1 or anti-hPD-L2. The T cell response curve in the presence of each antibody was depicted by a 4-parameter logistic function. Each plot is the data of individual experiments. All data are representative of two independent experiments. Bars, 5 μm. Error bars, standard deviation (SD). Source data for **a**, **b**, **d** and **e** are provided as a Source Data file.

Supplementary Table

	Pembrolizumab	Nivolumab	Durvalumab	Atezolizumab (μg/ml)
EC_{50} (95% CI)	0.1055<br>0.08100 to 0.1370	0.1341<br>0.1120 to 0.1600	0.1354<br>0.1227 to 0.1496	0.1202<br>0.1081 to 0.1336

Supplementary Table 1.

EC_{50} of individual clones of anti-hPD-1 or anti-hPD-L1 required for binding to their epitopes.

EC_{50} were calculated from the affinity curves of pembrolizumab, nivolumab, durvalumab or atezolizumab depicted by flowcytometry data in **Supplementary Fig. 3c**.

	Pembrolizumab	Nivolumab	Durvalumab	Atezolizumab (μg/ml)
EC_{50} (95% CI)	0.09666<br>0.01105 to 0.8453	0.1855<br>0.02507 to 1.373	0.02124<br>0.006749 to 0.06685	0.04284<br>0.004529 to 0.4052

Supplementary Table 2.

EC_{50} of individual clones of anti-hPD-1 or anti-hPD-L1 required for cancelling the PD-1-mediated suppression of mIFNγ production from T cells.

EC_{50} were calculated by the dose-response curves from the experiments in **Supplementary Fig. 5e**.

	Pembrolizumab	Nivolumab	24F.10C12 (μg/ml)
EC_{25} (95% CI)	0.2037<br>0.1329 to 0.2943	0.6753<br>0.4820 to 0.9186	0.03346<br>0.008739 to 0.06530
EC_{50} (95% CI)	0.592<br>0.4315 to 0.8293	2.056<br>1.479 to 3.256	0.07832<br>0.03953 to 0.1343
EC_{75} (95% CI)	1.72<br>1.113 to 3.006	6.259<br>3.750 to 13.87	0.1833<br>0.09922 to 0.4135
EC_{90} (95% CI)	5<br>2.626 to 11.93	19.06<br>8.954 to 61.16	0.4291<br>0.1669 to 1.787
EC_{98} (95% CI)	25.92<br>9.431 to 104.2	106.1<br>33.30 to 614.5	1.593<br>0.3253 to 20.97

Supplementary Table 3.

EC_{25}, EC_{50}, EC_{75}, EC_{90} and EC_{98}, of pembrolizumab, nivolumab or 24F.10C12 required for the recovery from the PD-1-mediated suppression of IL-2 production from T cells.

The data were calculated by the dose-response curves from the experiments in **Fig. 5g**.
