# Supplementary material for: Evaluation of therapeutic PD-1 antibodies by an advanced single-molecule imaging system detecting human PD-1 microclusters
Source: Nat Commun. 2023 Jun 6;14:3157. doi: 10.1038/s41467-023-38512-7 (PMC10244369; doi:10.1038/s41467-023-38512-7)
Supplement: Supplementary file 3 — Description of Additional Supplementary Files [file 41467_2023_38512_MOESM3_ESM.pdf]

Description of Additional Supplementary Files

File name: Supplementary Movie 1

Description: Human PD-1 forms microclusters in the presence of human PD-L1

AND-Tg Pdcd1$^{-/-}$ CD4^{+} T cells were introduced by hPD-1–EGFP, plated onto an MCC88-103-prepulsed planar bilayer containing I-E^{k}–GPI and ICAM-1–GPI without (left) or with hPD-L1–GPI (right) and imaged by TIRF microscopy at every 2.5 s. Bars, 5 µm. A representative of two independent experiments is shown.
