# Supplementary material for: Evaluation of therapeutic PD-1 antibodies by an advanced single-molecule imaging system detecting human PD-1 microclusters
Source: Nat Commun. 2023 Jun 6;14:3157. doi: 10.1038/s41467-023-38512-7 (PMC10244369; doi:10.1038/s41467-023-38512-7)
Supplement: Supplementary file 5 — Reporting Summary [file 41467_2023_38512_MOESM5_ESM.pdf]

nature portfolio

Corresponding author(s): Tadashi Yokosuka

Last updated by author(s): Apr 6, 2023

Reporting Summary

Nature Portfolio wishes to improve the reproducibility of the work that we publish. This form provides structure for consistency and transparency in reporting. For further information on Nature Portfolio policies, see our Editorial Policies and the Editorial Policy Checklist.

Statistics

For all statistical analyses, confirm that the following items are present in the figure legend, table legend, main text, or Methods section.

n/a	Confirmed	
☐	☒	The exact sample size (*n*) for each experimental group/condition, given as a discrete number and unit of measurement
☐	☒	A statement on whether measurements were taken from distinct samples or whether the same sample was measured repeatedly
☐	☒	The statistical test(s) used AND whether they are one- or two-sided<br>*Only common tests should be described solely by name; describe more complex techniques in the Methods section.*
☐	☒	A description of all covariates tested
☐	☒	A description of any assumptions or corrections, such as tests of normality and adjustment for multiple comparisons
☐	☒	A full description of the statistical parameters including central tendency (e.g. means) or other basic estimates (e.g. regression coefficient) AND variation (e.g. standard deviation) or associated estimates of uncertainty (e.g. confidence intervals)
☐	☒	For null hypothesis testing, the test statistic (e.g. *F*, *t*, *r*) with confidence intervals, effect sizes, degrees of freedom and *P* value noted<br>*Give P values as exact values whenever suitable.*
☒	☐	For Bayesian analysis, information on the choice of priors and Markov chain Monte Carlo settings
☒	☐	For hierarchical and complex designs, identification of the appropriate level for tests and full reporting of outcomes
☐	☒	Estimates of effect sizes (e.g. Cohen's *d*, Pearson's *r*), indicating how they were calculated

Our web collection on statistics for biologists contains articles on many of the points above.

Software and code

Policy information about availability of computer code

Data collection	FACS Canto II (BD, 07B1X00003000102), Guava easyCyte (MERCK, 0500-5007JPK), Prism v9.0.1 (GraphPad), FlowJo v10.5.0 (TreeStar)
Data analysis	Prism v9.0.1 (GraphPad), FlowJo v10.5.0 (TreeStar), Fiji v2.0

For manuscripts utilizing custom algorithms or software that are central to the research but not yet described in published literature, software must be made available to editors and reviewers. We strongly encourage code deposition in a community repository (e.g. GitHub). See the Nature Portfolio guidelines for submitting code & software for further information.

Data

Policy information about availability of data

All manuscripts must include a data availability statement. This statement should provide the following information, where applicable:

- Accession codes, unique identifiers, or web links for publicly available datasets
- A description of any restrictions on data availability
- For clinical datasets or third party data, please ensure that the statement adheres to our policy

All data supporting the conclusions included in the manuscript are available within the paper and its supplementary information. Source data are provided with this paper.

Human research participants

Policy information about studies involving human research participants and Sex and Gender in Research.

Reporting on sex and gender	n/a.
Population characteristics	n/a.
Recruitment	n/a.
Ethics oversight	n/a.

Note that full information on the approval of the study protocol must also be provided in the manuscript.

Field-specific reporting

Please select the one below that is the best fit for your research. If you are not sure, read the appropriate sections before making your selection.

☒ Life sciences ☐ Behavioural & social sciences ☐ Ecological, evolutionary & environmental sciences

For a reference copy of the document with all sections, see nature.com/documents/nr-reporting-summary-flat.pdf

Life sciences study design

All studies must disclose on these points even when the disclosure is negative.

Sample size	Sample size was selected based on similar studies previously performed and published, and not predetermined by a statistical method.
Data exclusions	No data were excluded form the analyses.
Replication	All experiments were repeated at indicated time in each figure legend and the results were repeatable.
Randomization	All cells were randomly selected in the analysis of imaging data.<br>Mice were randomly divided into experimental groups while assuring age and gender balance.
Blinding	The experimentalists were blinded during data collection and data analysis.

Reporting for specific materials, systems and methods

We require information from authors about some types of materials, experimental systems and methods used in many studies. Here, indicate whether each material, system or method listed is relevant to your study. If you are not sure if a list item applies to your research, read the appropriate section before selecting a response.

Materials & experimental systems

n/a	Involved in the study	
☐	☒	Antibodies
☐	☒	Eukaryotic cell lines
☒	☐	Palaeontology and archaeology
☐	☒	Animals and other organisms
☒	☐	Clinical data
☒	☐	Dual use research of concern

Methods

n/a	Involved in the study	
☒	☐	ChIP-seq
☐	☒	Flow cytometry
☒	☐	MRI-based neuroimaging

Antibodies

Antibodies used	The antibodies and reagents were purchased as follows: anti-IL-2 (1:500, JES6-1A12, e-Bioscience, 14-7022-85, RRID:AB_468406), biotin-labeled anti-IL-2 (1:1000, JES6-5H4, e-Bioscience, 13-7021-85, RRID:AB_466899), PE–anti-I-A/I-E (2.5 µg/ml, M5/114.15.2, BioLegend, 107608, RRID:AB_313323), PE–anti-mPD-1 (2.5 µg/ml, 29F.1A12, BioLegend, 135206, RRID:AB_1877231), anti-mPD-1 (2.5 µg/ml, 29F.1A12, BioLegend, 135248, RRID:AB_2783091), anti-hPD-L1 (2.5 µg/ml, 29E.2A3, BioLegend, 329746, RRID:AB_2783199), and anti-hPD-L2 (2.5 µg/ml, 24F.10C12, BioLegend, 329624, RRID:AB_2819957), PE–anti-hPD-1 (10 µl, MIH4, BD bioscience, 557946, RRID:AB_647199), PE–anti-hPD-L1 (10 µl, MIH1, BD bioscience, 557924, RRID:AB_647198), PE–anti-hPD-L2 (10 µl, MIH18, BD bioscience, 558066, RRID: AB_647197), Alexa Fluor 647–labeled anti-pCD3z (2.5 µg/ml, K25-407.69, BD bioscience, 558489, RRID:

AB_647152), and Alexa Fluor 647–labeled anti-pSLP-76 (2.5 µg/ml, J141-668.36.58, BD bioscience, 558438, RRID:AB_647159), rabbit polyclonal anti-SHP1 (1:500, C-19, Santa Cruz Biotechnology Inc., sc-287, RRID:AB_2173829) and mouse anti-SHP2 (1:1000, B-1, Santa Cruz Biotechnology Inc., sc-7384, RRID:AB_ 628252), anti-Erk (1:1000, Cell Signaling Technology, 4695S, RRID: AB_390779), anti-pErk (1:1000, Cell Signaling Technology, 4370S, RRID: AB_2315112), anti-PLC? (1:1000, Cell Signaling Technology, 5690S, RRID: AB_10691383), anti-pPLCg (1:1000, Cell Signaling Technology, 8713S, RRID: AB_10890863), anti-Akt (1:2000, Cell Signaling Technology, 4691S, RRID: AB_915783), anti-pAkt (1:1000, Cell Signaling Technology, 4060S, RRID: AB_2315049), and HRP-anti-rabbit IgG polyclonal Abs (1:10000, Cell Signaling Technology, 7074S, RRID: AB_2099233), HRP-anti-mouse IgG polyclonal Abs (1:10000, Cappel, 55550), pembrolizumab (MCE, HY-P9902), nivolumab (MCE, HY-P9903), durvalumab (MCE, HY-P9919), and atezolizumab (MCE, HY-P9904), APC-anti-human IgG (H+L) (2.5 µg/ml, Jackson Immuno Research, 705-136-147, RRID: AB_2340407). A B cell hybridoma producing anti-CD28 (PV-1) was provided by R. Abe (Tokyo University of Science, Noda, Japan); anti-CD3z (145-2c11) by J. Bluestone (University of California, San Francisco, USA); anti-TCRβ (H57-597) by R. T. Kubo (Cytel Corp., CA, USA); and anti-I-Ek (14-4-4) and anti-ICAM-1 (YN1/1.7.4) by M. L. Dustin (University of Oxford, UK); anti-hPD-L1 (MIH1) and anti-hPD-L2 (MIH18) by M. Azuma (Tokyo Medical and Dental University, Tokyo, Japan).

Validation

Validation statements

e-Bioscience-Thermo Fisher Scientific:
"Thermo Fisher Scientific is committed to adopting higher validation standards for the Invitrogen antibody portfolio. We have implemented additional specificity tests to help ensure the highest confidence levels in our products. You can identify the products that have already undergone this testing with the Advanced Verification badge, shown above. This badge can be found in antibody search results and at the top of product webpages. The data supporting the Advanced Verification status can be found in the product specific data galleries. To learn more about our testing standards, please visit Invitrogen Antibody Validation."

BioLegend:
"Clones of these hybridomas are carefully selected based on a number of criteria including robust growth and efficient production of a single clone of antibody that is specific to the intended target. The best clones move on to applications testing.
The steps include:
Immunogen design and construction
Immunization of host animal
Hybridoma creation
ELISA or application-specific screening of antibodies from clones
Application testing, including WB, ELISA, ChIP, IF, IHC, or biofunctional assays"

BD Biosciences:
"The specificity is confirmed using multiple methodologies that may include a combination of flow cytometry, immunofluorescence, immunohistochemistry or western blot to test staining on a combination of primary cells, cell lines or transfectant models.
All flow cytometry reagents are titrated on the relevant positive or negative cells. To save time and cell samples for researchers, test size reagents are bottled at an optimal concentration with the best signal-to-noise ratio on relevant models during the product development. To ensure consistent performance from lot-to-lot, each reagent is bottled to match the previous lot MFI. You can look up the Certificate of Analysis and the concentration of test-size human reagents from specific lots via the Concentration Lookup page or BD Regulatory Documents."

Cell Signaling Technology:
"At Cell Signaling Technology (CST), we understand that there is no single assay that can determine the validity of an antibody. Confirming that an immunoreagent is sufficiently specific and sensitive depends on the application and protocol being used, the type and quality of sample being analyzed, and the inherent biophysical properties of the antibody itself.
To ensure our antibodies will work in your experiment, we adhere to the Hallmarks of Antibody Validation™, six complementary strategies that can be used to determine the functionality, specificity, and sensitivity of an antibody in any given assay. CST adapted the work by Uhlen, et. al., (“A Proposal for Validation of Antibodies.” Nature Methods (2016)) to build the Hallmarks of Antibody Validation, based on our decades of experience as an antibody manufacturer and our dedication to reproducible science."

Eukaryotic cell lines

Policy information about cell lines and Sex and Gender in Research

Cell line source(s)

The DC-1 fibroblast cell expressing I-Ek and ICAM-1 was provided by J. Kaye (Cedars-Sinai Medical Center, Los Angeles, CA). PLAT-E, the retrovirus packaging cell line, was provided by G. Nolan (Stanford University, Stanford, CA; Cell Biolabs, RV-101). Human lung cancer cell lines H460, H1299, and HCC827 were purchased from ATCC (ATCC, NCI-H460, RRID:CVCL_0459; ATCC, NCI-H1299, RRID:CVCL_0060; ATCC, HCC827 PFR1, RRID:CVCL_DH92). BHK, EL-4, and E. G7-OVA Cell Line were purchased from ATCC (ATCC, ACC-61, RRID:CVCL_1915; ATCC, TIB-39, RRID:CVCL_0255; ATCC, CRL-2113, RRID:CVCL_3505). The T cell hybridoma expressing the AND-TCR (AND-TCR T cell hybridoma, 2D12) was established by cell fusion of activated AND TCR-Tg CD4+ T cells with lymphoma cell line, BW514726.

Authentication

DC-1 and PLAT-E were not authenticated.

Mycoplasma contamination

All cell lines were tested negative for Mycoplasma contamination.

Commonly misidentified lines
(See ICLAC register)

None.

Animals and other research organisms

Policy information about studies involving animals; ARRIVE guidelines recommended for reporting animal research, and Sex and Gender in Research

Laboratory animals	AND TCR-Tg mice were provided by Dr. S. M. Hedrick (University of California San Diego, San Diego, CA); Rag2-/- mice by Dr. F. Alt (Boston Children's Hospital, Boston, MA); OT-I TCR-Tg Rag2-/- mice by Dr. W. Health (University of Melbourne, Melbourne, Australia); and Pdcd1-/- mice from RIKEN BRC. Mice were maintained in specific pathogen-free conditions with a 12 h light/dark cycle at 22 °C and controlled humidity (60±10%) at Tokyo Medical University. All experiments were performed on 6-10 weeks old mice, age- and gender-matched. Experimental and control animals were co-housed.
Wild animals	No wild animals were used in the study.
Reporting on sex	All experiments were performed on 6-10 weeks old mice, age- and gender-matched. Gender was not related to the results.
Field-collected samples	No field collected samples were used in the study.
Ethics oversight	All experiments were performed in accordance with a protocol approved by the Animal Care and Use Committee of Tokyo Medical University (H30-0044, H31-0065, R2-0001).

Note that full information on the approval of the study protocol must also be provided in the manuscript.

Flow Cytometry

Plots

Confirm that:

☒ The axis labels state the marker and fluorochrome used (e.g. CD4-FITC).

☒ The axis scales are clearly visible. Include numbers along axes only for bottom left plot of group (a 'group' is an analysis of identical markers).

☒ All plots are contour plots with outliers or pseudocolor plots.

☒ A numerical value for number of cells or percentage (with statistics) is provided.

Methodology

Sample preparation	The cells were suspended with FACS buffer (2% FCS/PBS) and stained with fluorescence-conjugated antibodies. Stained cells were washed twice with FACS buffer and then analazed.
Instrument	Guava easyCyte (MERCK), Canto II (BD), SH800 (Sony)
Software	FlowJo v10.5.0
Cell population abundance	100000 events in FSC/SSC parameters were measured to analyze.
Gating strategy	Live cells were gated by FSC/SSC and doublet cells were removed. Then target cells were counted or sorted for fluorescence-positive area.

☒ Tick this box to confirm that a figure exemplifying the gating strategy is provided in the Supplementary Information.
